# Quetiapine Inhibits Microglial Activation by Neutralizing Abnormal STIM1-Mediated Intercellular Calcium Homeostasis and Promotes Myelin Repair in a Cuprizone-Induced Mouse Model of Demyelination

**DOI:** 10.3389/fncel.2015.00492

**Published:** 2015-12-21

**Authors:** Hanzhi Wang, Shubao Liu, Yanping Tian, Xiyan Wu, Yangtao He, Chengren Li, Michael Namaka, Jiming Kong, Hongli Li, Lan Xiao

**Affiliations:** ^1^Chongqing Key Laboratory of Neurobiology, Department of Histology and Embryology, Third Military Medical University, Chongqing, China; ^2^College of Pharmacy and Medicine, Joint Laboratory of Biological Psychiatry Between Shantou University Medical College and College of Medicine, University of Manitoba, Winnipeg, MB, Canada; ^3^Department of Human Anatomy and Cell Science, College of Medicine, University of Manitoba, Winnipeg, MB, Canada; ^4^Department of Rehabilitation Medicine, Health Sciences Centre (HSC), Winnipeg, MB, Canada

**Keywords:** quetiapine, microglia, calcium homeostasis, stored-operated calcium entry, stromal interaction molecule 1

## Abstract

Microglial activation has been considered as a crucial process in the pathogenesis of neuroinflammation and psychiatric disorders. Several antipsychotic drugs (APDs) have been shown to display inhibitory effects on microglial activation *in vitro*, possibly through the suppression of elevated intracellular calcium (Ca^2+^) concentration. However, the exact underlying mechanisms still remain elusive. In this study, we aimed to investigate the inhibitory effects of quetiapine (Que), an atypical APD, on microglial activation. We utilized a chronic cuprizone (CPZ)-induced demyelination mouse model to determine the direct effect of Que on microglial activation. Our results showed that treatment with Que significantly reduced recruitment and activation of microglia/macrophage in the lesion of corpus callosum and promoted remyelination after CPZ withdrawal. Our *in vitro* studies also confirmed the direct effect of Que on lipopolysaccharide (LPS)-induced activation of microglial N9 cells, whereby Que significantly inhibited the release of nitric oxide (NO) and tumor necrosis factor α (TNF-α). Moreover, we demonstrated that pretreatment with Que, neutralized the up-regulation of STIM1 induced by LPS and declined both LPS and thapsigargin (Tg)-induced store-operated Ca^2+^ entry (SOCE). Finally, we found that pretreatment with Que significantly reduced the translocation of nuclear factor kappa B (NF-κB) p65 subunit from cytoplasm to nuclei in LPS-activated primary microglial cells. Overall, our data suggested that Que may inhibit microglial activation by neutralization of the LPS-induced abnormal STIM1-mediated intercellular calcium homeostasis.

## Introduction

Microglia represents an abundant portion of cells that comprise the central nervous system (CNS). They are exclusively distributed in brain and spinal cord and represent about 5–20% of the total glial cell population (Lawson et al., 1990). CNS microglia cells are resident immune cells of the brain that constantly monitor the cerebral microenvironment to resist pathogens and heal injuries (Perry et al., 1993). Recent research demonstrates that microglial activation has a critical role in pathogenesis of neuroinflammatory diseases such as multiple sclerosis (MS) (Jack et al., 2005), ­neurodegenerative diseases, such as Alzheimer’s disease (AD) (Wang et al., 2015) or Parkinson’s disease (PD) (Qian et al., 2010), by producing various proinflammatory cytokines and free radicals (Kettenmann et al., 2011; Smith and Dragunow, 2014; Streit et al., 2014; Probert, 2015). In addition, other studies have also linked the importance of microglial activation to the pathogenesis associated with schizophrenia (SZ) (Monji et al., 2009; Busse et al., 2012; Monji et al., 2013; Al-Hakeim et al., 2015; An et al., 2015). Specifically, it has been shown that an elevated microglial density or microglial activation has been observed in the brains of patients with SZ (Steiner et al., 2008; Doorduin et al., 2009; Kato et al., 2013; Watkins and Andrews, 2015). Atypical antipsychotic drugs (APDs), such as risperidone, olanzapine, and aripiprazole have been reported to reduce the secretion of TNF-α and nitric oxide (NO) from activated microglia (Hou et al., 2006; Bian et al., 2008; Kato et al., 2008). These studies suggested that the pharmacological action of the antipsychotics on microglia may underlie the reported benefits associated with the use of these agents in patients with SZ (Kato et al., 2011).

Recently, accumulating evidence points to oligodendroglia dysfunction in regard to the demyelination known to be involved in the pathogenesis of SZ. As such, drugs that target oligodendroglia function are being investigated for their potential benefit in SZ (Ren et al., 2013; Roussos and Haroutunian, 2014). Que is an atypical APD that has been demonstrated to have superior therapeutic effects on cognitive symptoms displayed by patients with SZ as well as other neurological disorders (Kasper and Muller-Spahn, 2000; Riedel et al., 2007). It has been found that Que can protect mice from CPZ-induced microglial activation and myelin breakdown (Zhang et al., 2008; Shao et al., 2015). Que has also been shown to modulate immune responses in an experimental autoimmune encephalomyelitis (EAE) model of MS (Mei et al., 2012). It has also been shown to inhibit release of proinflammatory factors from activated microglia in culture (Bian et al., 2008). However, the underlying mechanism by which Que regulates microglial activation remains elusive. Meanwhile, it is also unclear as to the specific actions of Que on microglial activation during remyelination.

Interestingly, research has suggested that the elevation of intracellular calcium (Ca^2+^) is critical in cell proliferation, migration, or ramification (Mizoguchi et al., 2014). Previous studies reported that pretreatment with APDs (Kato et al., 2008; Mizoguchi et al., 2014) significantly inhibited the release of proinflammatory cytokines and/or NO from activated microglia by suppression of elevation of intracellular calcium concentration ([Ca^2+^]_*i*_). Henceforth, it is possible that Que may also inhibit microglial activation *via* suppression of [Ca^2+^]*_*i*_* elevation; however, the molecular pathway for this proposed effect is yet to be defined. Among Ca^2+^ regulation in non-excitable cells, the main Ca^2+^ influx mechanism is called store-operated Ca^2+^ entry (SOCE) (Hoffmann et al., 2003; Qian et al., 2010; Kettenmann et al., 2011; Brawek and Garaschuk, 2013; Heo et al., 2015). Studies also reported that Ca^2+^ release from SOCE stimulates an intercellular proinflammatory signal (Ohana et al., 2009; Mizoguchi et al., 2011), indicating that SOCE may contribute to the release of proinflammatory substances during microglial activation (Kraft, 2015; Michaelis et al., 2015; Moccia et al., 2015). However, to the best of our knowledge, there is currently no study that has addressed this issue.

In the present study, using a CPZ-induced chronic demyelination mouse model, as well an *in vitro* systems using lipopolysaccharide (LPS)-induced activated microglial, we demonstrated that Que dramatically attenuated microglial activation and promoted myelin repair. We also found that Que can neutralize the STIM1-mediated elevation of Ca^2+^ entry (SOCE) and subsequent NFκB activation in LPS-induced activated microglia.

## Materials and Methods

### Animals and Experimental Manipulations

C57BL/6 mice (male, 6 weeks old, 22–25 g) were obtained from the Animal Facility Centre of the Third Military Medical University, PR China. The animals were housed at this facility with a 12-h dark/12-h light cycle, at a constant temperature of 22 ± 1°C and a relative humidity of 60%. All procedures were performed in accordance with the guidelines set and approved by the Laboratory Animal Welfare and Ethics Committee of the Third Military Medical University.

C57BL/6 mice were randomly assigned to one of the following four groups: control (*CTL*), in which mice fed regular chow and drank distilled water for 12 weeks; *CPZ*, in which mice fed 0.2% CPZ for 12 weeks to induce a chronic demyelination (Matsushima and Morell, 2001); *Veh*, in which mice fed 0.2% CPZ for 12 weeks, then fed regular chow, and drank vehicle water for 2 weeks; *Que*, in which mice fed 0.2% CPZ for 12 weeks, and then fed regular chow, and drank Que-containing water for 2 weeks.

### Drug Treatments

Cuprizone (bis-cyclohexanone oxaldihydrazone) was purchased from Sigma-Aldrich (St. Louis, MO, USA). As previously described in other studies, 0.2% CPZ was mixed into the ground standard rodent chow. Que was provided by Astra Zeneca (Wilmington, DE, USA) and dissolved in distilled water. The mice ingested 10 mg/kg/day of Que according to our previously established in house methods with this model (Xiao et al., 2008; Zhang et al., 2008).

Lipopolysaccharide (*Escherichia coli*, E5:055) was purchased from Sigma (St. Louis, MO, USA). Fetal bovine serum (FBS) was purchased from Hyclone (Logan, UT, USA). Thiazolylblue (MTT) was from Beyotime Institute of Biotechnology, China (Shanghai, China). Iscove’s Modified Dulbecco’s medium (IMDM) was from Hyclone (Logan, UT, USA) and Dulbecco’s Modified Eagle Medium (DMEM) was from Gibco (Life technologies).

Que was dissolved into 20 mM of dimethyl sulfoxide (DMSO) and then diluted into 2 mM of PBS for experiments. The concentrations of Que were 0.1, 1, 10, and 50 μM.

### Histology and Immunohistochemistry

Histology and immunohistochemistry were performed as previously described (Wang et al., 2010). Briefly, 20 μm serial frozen sections between bregma −0.94 and bregma −1.8 [according to mouse atlas by Paxinos and Franklin (2004)] were analyzed. Sections were stained for myelin with Luxol fast blue (LFB)/periodic acid-Schiff (PAS) base. For immunohistochemistry, sections were quenched with H_2_O_2_ blocked for 30 min in PBS containing 3% bovine serum albumin, 0.1% Triton X-100, and then incubated overnight with primary antibody. The primary antibody myelin basic protein (MBP) (Goat IgG; Santa Cruz) was used as a myelin protein. After washing, sections were further incubated with biotinylated secondary antibody (Daco) for 1 h, followed by peroxidase-coupled avidin–biotin complex (ABC kit, Vector Laboratories).

### N9 Cell Culture

The murine microglial cell line N9 (Provided by Prof. Yun Bai, Department of Medical Genetics, TMMU, China) were grown in DMEM supplemented with 10% heat-inactivated FBS, 100 U/ml penicillin, 100 μg/ml streptomycin, and 2 mM glutamine in humidified atmosphere of 95% air and 5% CO_2_ at 37°C. The medium was changed every 2 days. Cells were plated at a density of 4 × 10^4^ cells/well onto 96-well microtiter plates for MTT and nitrite assay. Que with or without LPS (100 ng/ml) was added to the culture medium of N9 cells for 24 h.

### Primary Microglial Cell Culture

Primary microglia cultures from C57BL/6 mice, cells were prepared from postnatal days 1–3. The brain tissue was dissociated in ice-cold HBSS. After removal of the cerebellum and subcortical tissue, meninges and blood vessels were dissociated under a dissecting microscope. The cortex was placed in an additional petri dish with precooled DMEM/F12 medium. The petri dish was placed on ice. The cerebral cortex was cut into about 1 mm^3^ blocks, digested in 0.125% trypsin at 37°C for 5 min, and agitated into a cell suspension. Cell aggregates were collected by centrifugation (1000 × *g*, 5 min), resuspended in DMEM/F12, containing 10% FBS and antibiotics (40 U/ml penicillin and 40 μg/ml streptomycin) and cultured in 95% air and 5% CO_2_ at 37°C. Floating microglia were harvested every week (between 2 and 7 weeks) and reseeded into 75 cm^2^ culture flask to give pure microglia cultures. The medium was replaced once every 3 days.

### MTT Assay

Cell viability was evaluated by the MTT reduction assay as described previously (Niu et al., 2010). The cells were seeded in a 96-well plate for 24 h before being exposed to Que alone (10 μm) or Que with LPS (100 ng/ml) for 24 h. MTT solution (0.5 mg/ml, Beyotime, Nantong, China) was then added to each well and the cells were incubated for 1 h at 37°C and in 5% CO_2_. Subsequently, the supernatant was removed and the formation of farmazan was solubilized with DMSO and measured at 540 nm with SpectraMax M2e spectrophotometer (Molecular Devices, Sunnyvale, CA, USA).

### Nitrite Production Assessment

Accumulation of nitrite (NO_2_^−^) in the culture media, an indicator of NO synthase activity, was measured by Griess Reaction. Cells at density of 3 × 10^4^ cells/well were plated onto 96-well microtiter plates. Que with or without LPS (100 ng/ml) were added to the culture medium of N9 microglial cells for 48 h. Fifty microliters of culture supernatants were mixed with 50 μl Griess reagents (Part I: 1% sulfanilamide; Part II: 0.1% naphthylethylene diamide dihydrochlride and 2% phosphoric acid) at room temperature at 540 nm using the microplate reader. Nitrite concentration was calculated with reference to a standard curve of sodium nitrite.

### Immunofluorescent Staining

Cells on glass cover slips were fixed with 4% paraformaldehyde (PFA), rinsed with 0.01M PBS, incubated with 0.3% TritonX-100 for 5 min and blocked in 3% BSA for 60 min. Then glass cover slips were incubated in following primary antibodies overnight at 4°C: for NFκB p65 (rabbit IgG; Santa Cruz), for microglia CD11b (mouse IgG; Chemico), washed with PBS, and incubated with fluorescence-conjugated second antibodies at 4°C overnight. The method used for brain sections has been described previously (Wang et al., 2010). The immunoreactivity was determined using a 20× objective lens on a fluorescence microscope (Olympus BX-60) and a TCS SP5 confocal laser scanning microscope (Leica) with an excitation wavelength appropriate for 488 or 528 nm. Cell nuclei were stained with DAPI (Sigma, 0.1 μg/ml in PBS) at room temperature for 15 min. Cell counting was conducted on nine randomly chosen fields for each cover slip by using the densitometer Image Pro Plus image analysis system. There were two cover slips in each group.

### Intracellular Ca^2**+**^ Imaging

Microglial N9 cells were plated at 1 × 10^6^ cells on poly-d-lysine-coated, glass-bottomed culture dishes. Cells were incubated in medium containing 2 μM Fura-3 for 15 min at 37°C. Before Ca^2+^ measurements were conducted, the culture dishes were washed with Ca^2+^-free standard extracellular solution (SES) buffer. Cells were incubated with medium alone and 50 nM Que for 20 min before addition of LPS or thapsigargin (Tg). Tg is non-competitive inhibitor of the sarco/endoplasmic reticulum (ER) Ca^2+^ ATPase (SERCA). During fluorescent measurements, the cells were continually perfused with a regular solution (37°C) containing 150 mM NaCl, 5 mM KCl, 1 mM MaCl_2_, 10 mM glucose, and 10 mM HEPES at pH 7.4 with NaOH and either 1–2 mM CaCl_2_ or 0.5 mM EGTA (Ca^2+^-free). Fluorescent measurements were performed by imaging the Fluo-3 AM-loaded microglia using a laser scanning confocal microscope (Olympus IV 1000). Images were acquired using an olympus fluoview Ver.2.1c Viewer software. Relative average intracellular Ca^2+^ concentration values were obtained from at least 20–30 microglial cells and the results obtained from at least three or four individual experiments.

### Quantitative RT-PCR

RNA was isolated from cultures using TRIzol Reagent (Invitrogen) and total RNA (5 mg) was reverse transcribed using PrimeScript™ RT-PCR Kit (Takara) according to the manufacturer’s instructions. The cDNA was analyzed by real-time PCR with the Rotor Gene6000 (Corbertt Research, Australia) according to the protocol provided by the manufacturer and 2^−ΔΔCt^ method. Briefly, PCRs were performed using SYBR premix Ex Taq (Takara) in a final volume of 20 μl. The specific primers of target genes were as follows: TNF-α (5′-GACGTGGAACTGGCAGAAGAG-3′, 5′-TGCCACAAGCAGGAATGAGA-3′), Stim1 (5′-TCTFCATGACCTTCAGGAAA-3′, 5′-GGTGGACCTTCTCTACTTCCAC-3′), Stim2 (5′-AGTTGCCCTGCTCTGTATCG-3′, 5′-TGAAGCTGTTGTCTGGCACTT-3′), Orai1 (5′-TACTTAAGCCGCGCCAAG-3′, 5′-ACTTCCACCATCGCTACCA-3′), and GAPDH (5′-CAGCAAGGACACTGAGCAAGA-3′, 5′-GCCCCTCCTGTTATTATGGGG-3′).

### Statistical Analysis

One-way or two-way analysis of variance (ANOVA) was used to test statistical significance of three or more experimental groups, which was followed by Dunnett’s *post hoc* or Tukey’s *post hoc* test. Comparison between two experimental groups was made by the Student’s *t*-test. A probability of *P* < 0.05 was considered ­statistically significant.

## Results

### Que Inhibits the Activation of Microglia/Macrophage in Corpus Callosum Lesions

**Figure 1:**
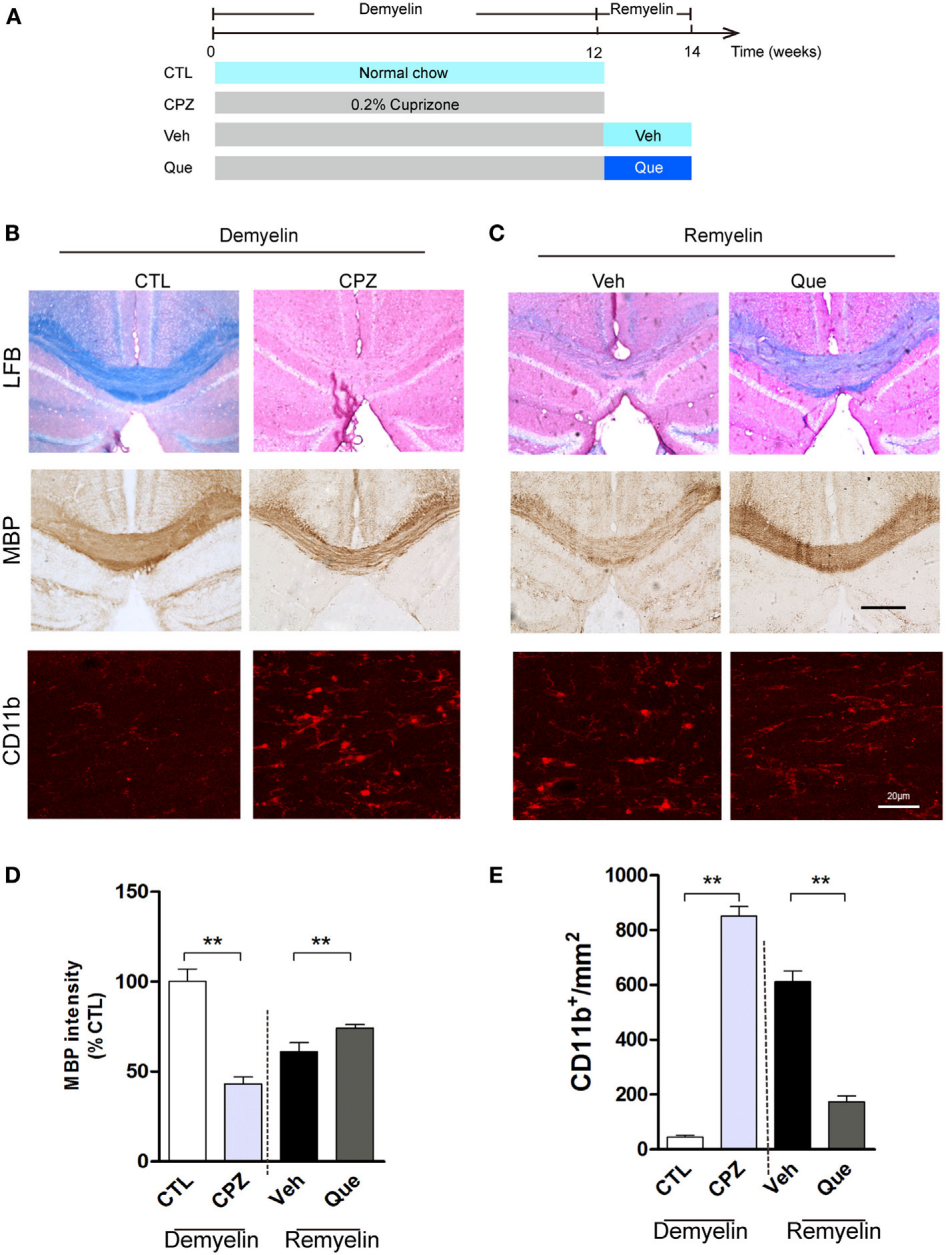
**Effect of Que on microglial activation and remyelination in CPZ-induced chronic demyelinating mouse model**. **(A)** Schematic diagram displaying the time course and Que (10 mg/kg/day) treatment on CPZ-induced demyelination and remyelination. C57BL/6 mice were given CPZ for 12 weeks for chronic demyelination and start remyelination after CPZ withdrawal. **(B)** Representative Luxol fast blue staining, MBP and CD11b IF staining for corpus callosum (CC) mediolateral area of mice fed with or without CPZ. **(C)** Representative Luxol fast blue staining, MBP and CD11b IF staining for CC mediolateral areas of mice fed with or without Que after CPZ withdraw. **(D)** Quantification for optical density of MBP expression in CC of indicated groups. **(E)** Quantification of CD11b^+^ cells (activated microglia) in CC. The scale bars are 200 μm and 20 μm, respectively. Data represent means ± SEM (*n* = 6 in each group), **p < 0.01 between indicated group.

To investigate the effect of Que on microglial activation involved in myelin defects, we used long-term CPZ-treated mice to mimic the neuroinflammation and white matter deterioration known to occur in the chronic disease phase (Figure 1A). Fast blue-staining results showed that almost no myelin fibers could be detected in corpus callosum (CC) in CPZ group vs. the control group (CTL) (Figure 1B). After CPZ withdrawal, remyelination was observed. However, the extent of myelin repair of CC was much higher in the Que group compared to that in the Veh group (Figure 1C). Similar demyelination and remyelination trends were observed in MBP-positive immunostaining (Figures 1B,C). Statistical analysis revealed a significant difference between CTL and CPZ group in terms of the optical density of MBP immunostaining, while Que group significantly increased in optical density of MBP staining compared to Veh group (Figure 1D). Interestingly, the accumulation of activated microglia/macrophages was observed by CD11b staining in the CC of CPZ group while only sporadically seen in CTL group (Figure 1B). After CPZ withdrawal, the density of CD11^+^ cells (active microglia) decreased slightly but still remained at an elevated level that was not seen in the Que group (Figures 1C,E). These data suggest that Que can alleviate the recruitment and activation of microglia and promote myelin repair in CPZ-induced chronic mouse model of demyelination.

### Que Decreases the Release of NO and TNF-α from Activated N9 Microglial Cells Induced by LPS

**Figure 2:**
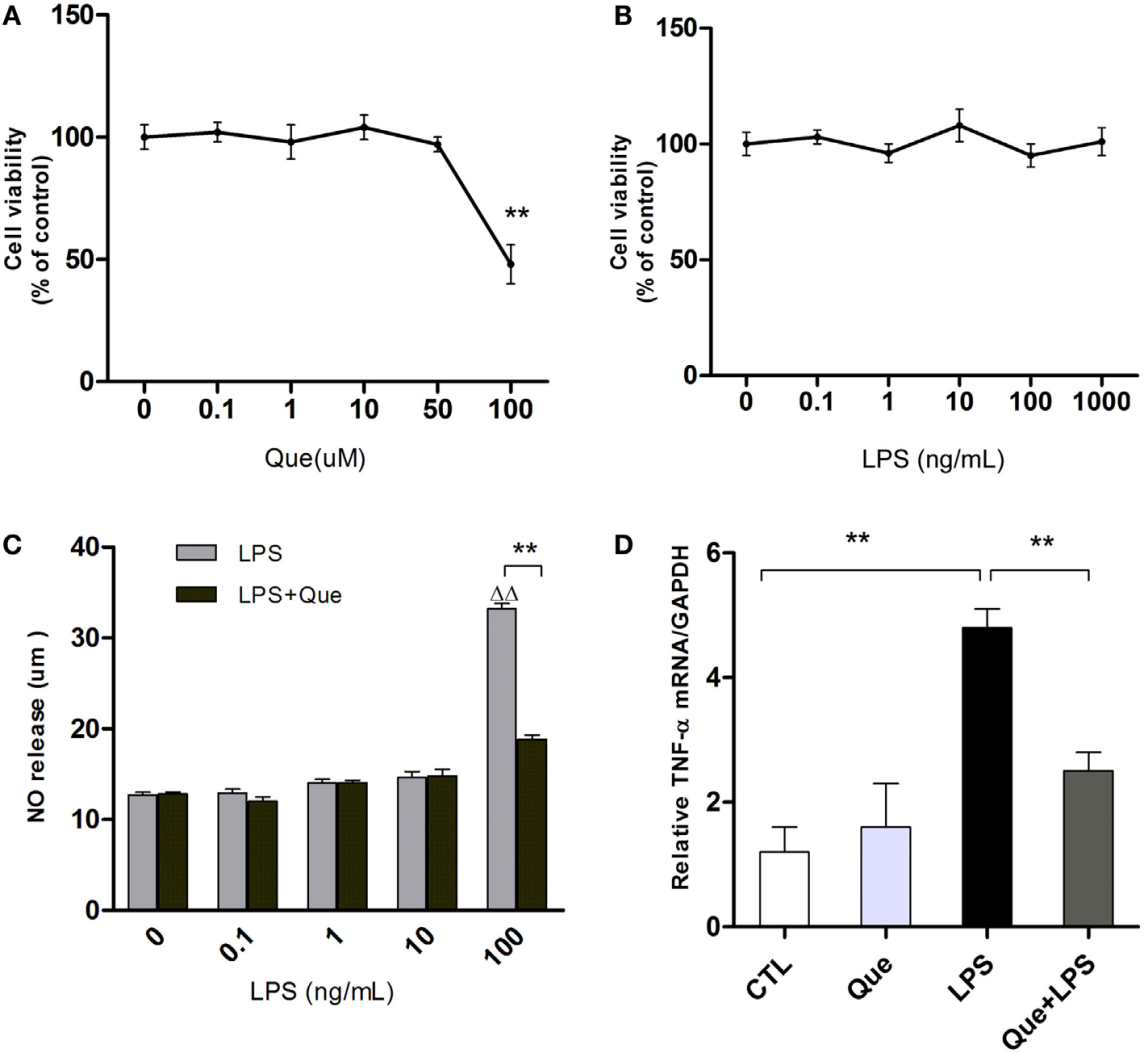
**Effect of Que on NO and TNF-α release from N9 cells induced by LPS**. **(A)** MTT assay, cell viability of N9 microglial cells treated with various concentrations (0, 0.1, 1, 10, 50, and 100 μM) of Que for 24 h. **(B)** MTT assay, cell viability of N9 microglial cells treated with various concentrations (0, 0.1, 1, 10, 100, and 1000 ng/ml) of LPS for 24 h. **(C)** Griess assay of NO release from N9 cells exposed to various concentrations of LPS for 24 h with or without Que (10 μM) pretreatment. **(D)** qRT-PCR measurement of TNF-α mRNA expression in N9 cells exposed to LPS (100 ng/ml) for 24 h with or without Que (10 μM) pretreatment. Data are represented as means ± SEM. **p < 0.01 vs. indicated group ^▵▵^ *p* < 0.01 vs. control group (LPS=0ng/mL).

To exclude non-specific effects of Que on microglial cells, MTT assay was performed to observe cell viabilities of N9 microglial cells treated with or without Que. Results showed that Que had no significant effect on cell viabilities at various concentrations under 100 μM, in which significant toxicity could be observed (Figure 2A). In addition, cell viability of N9 cells were tested after exposure to LPS at various concentrations (0, 0.1, 1, 10, 100, and 1000 ng/ml). The results displayed that LPS had no significant effect on cell viabilities (Figure 2B, *P* > 0.05). However, NO release in medium was increased by LPS in concentration-dependent manner, which was dramatically inhibited by pretreatment of Que (10 μM) (Figure 2C). Similar inhibitory effects of Que on TNF-α synthesis were also observed (Figure 2D). As such, our results demonstrated that Que did not affect N9 microglia cells viability, but decreased the release of NO and TNF-α from N9 microglial cells induced by LPS.

### Que Inhibits Ca^2**+**^ Elevation in N9 Cells Induced by LPS and Thapsigargin (Tg)

**Figure 3:**
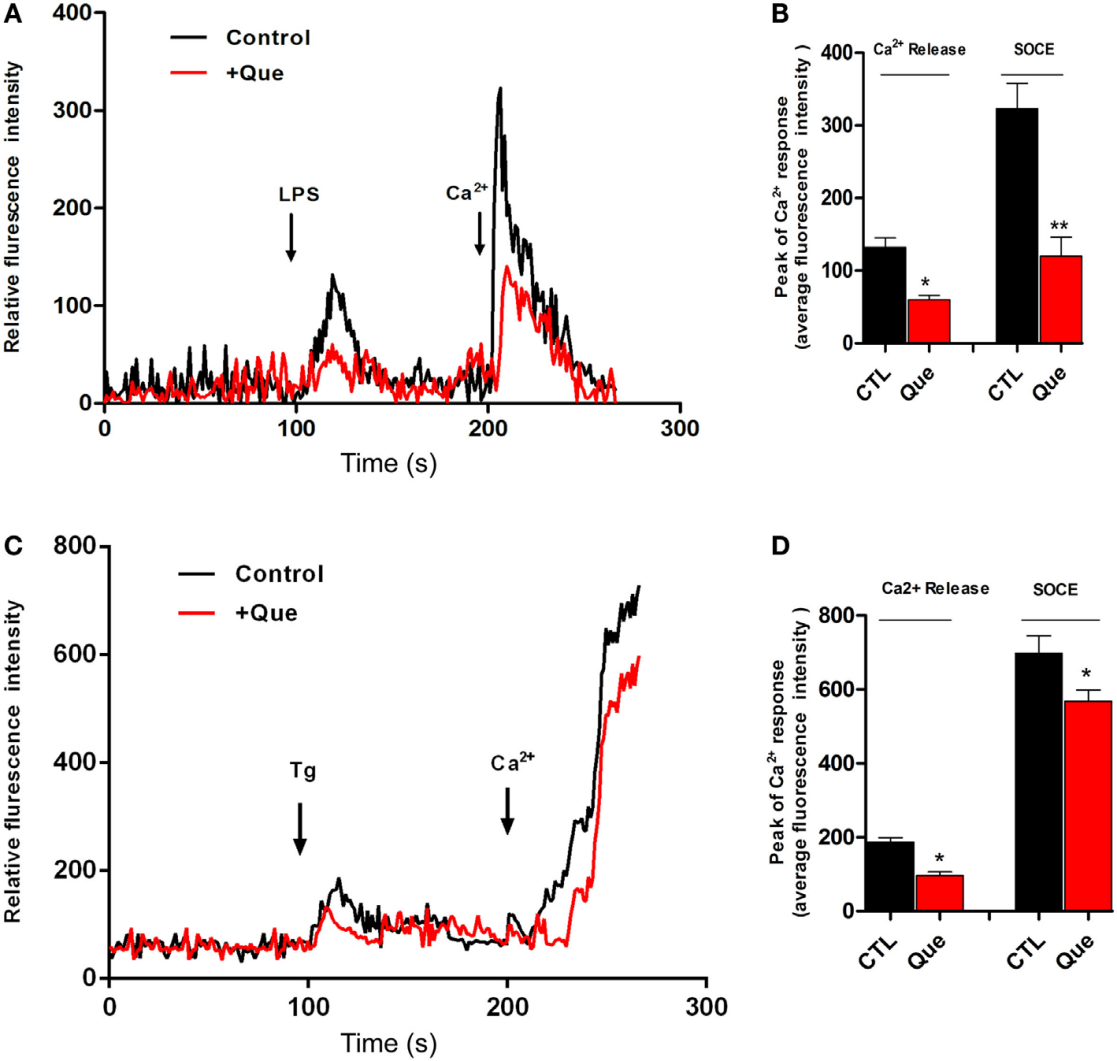
**Effect of Que on Ca^2+^ release and Ca^2+^ entry in N9 cells induced by LPS or Tg**. **(A)** Ca^2+^ image of N9 cells with LPS stimulation in Ca^2+^-free medium following by Ca^2+^ (2 mM) buffer incubation after wash with or without Que (10 μM) pretreatment. The first peak shows [Ca^2+^]*_i_* elevation due to release of internal ER Ca^2+^ induced by LPS; the second peak is due to Ca^2+^ influx through the PM, namely ER Ca^2+^ store-operated Ca^2+^ entry (SOCE). Pretreatment of Que reduces the [Ca^2+^]*_i_* elevation induced by LPS (red) (20–25 cells were analyzed in each group). **(B)** Quantification of Ca^2+^ release (left peak) and store-operated Ca^2+^ entry (SOCE) (right peak) in N9 cells activated by LPS with or without Que pretreatment. **(C)** Ca^2+^ image of N9 cells with Tg stimulation in Ca^2+^-free medium following by Ca^2+^ (2 mM) buffer incubation after wash with or without Que pretreatment (20–25 cells were analyzed in each group). **(D)** Quantification of Ca^2+^ release (left peak) and store-operated Ca^2+^ entry (SOCE) (right peak) in N9 cells activated by Tg with or without Que pretreatment. Values are means ± SEM. *p < 0.05 and **p < 0.01 vs. CTL.

To determine if Que might affect the Ca^2+^ signaling pathway, which is very important for microglial activation, we utilized Ca^2+^ imaging to measure alterations of [Ca^2+^]*_*i*_* in N9 cells after stimulation. It was found that LPS induced sustained [Ca^2+^]*_*i*_* elevation due to release of internal ER Ca^2+^ (left peak, arrow) in Ca^2+^-free imaging buffer. After washing, 2 mM Ca^2+^ buffer induced [Ca^2+^]*_*i*_* elevation (right peak, arrow) due to Ca^2+^ influx through the PM, namely SOCE (Figure 3A). Pretreatment of Que for 15 min showed a significant decrease in the [Ca^2+^]*_*i*_* level in response to LPS stimulation (Figure 3B). These results suggested that Que may reduce LPS-stimulated [Ca^2+^]*_*i*_* by inhibiting activation-induced Ca^2+^ channel in PM and Ca^2+^ influx. In addition, in order to verify whether Que affect SOCE in N9 cells, or to examine the specificity of Que effects on Ca^2+^ influx, we investigated the effect of Que on TG-induced activation of [Ca^2+^]*_*i*_*. Stimulation of Tg on N9 cells induced both Ca^2+^ release in ER (left peak in Figure 3C) and Ca^2+^ influx in PM (right peak in Figure 3C); pre-treated with Que, however, decreased TG-induced Ca^2+^ turn over (Figures 3C,D). Our results suggest that Que inhibits Ca^2+^ elevation likely by modulation SOCE in N9 microglial cells induced by LPS or Tg.

### Que Inhibits Upregulation of STIM1 in N9 Cells Exposed to LPS

**Figure 4:**
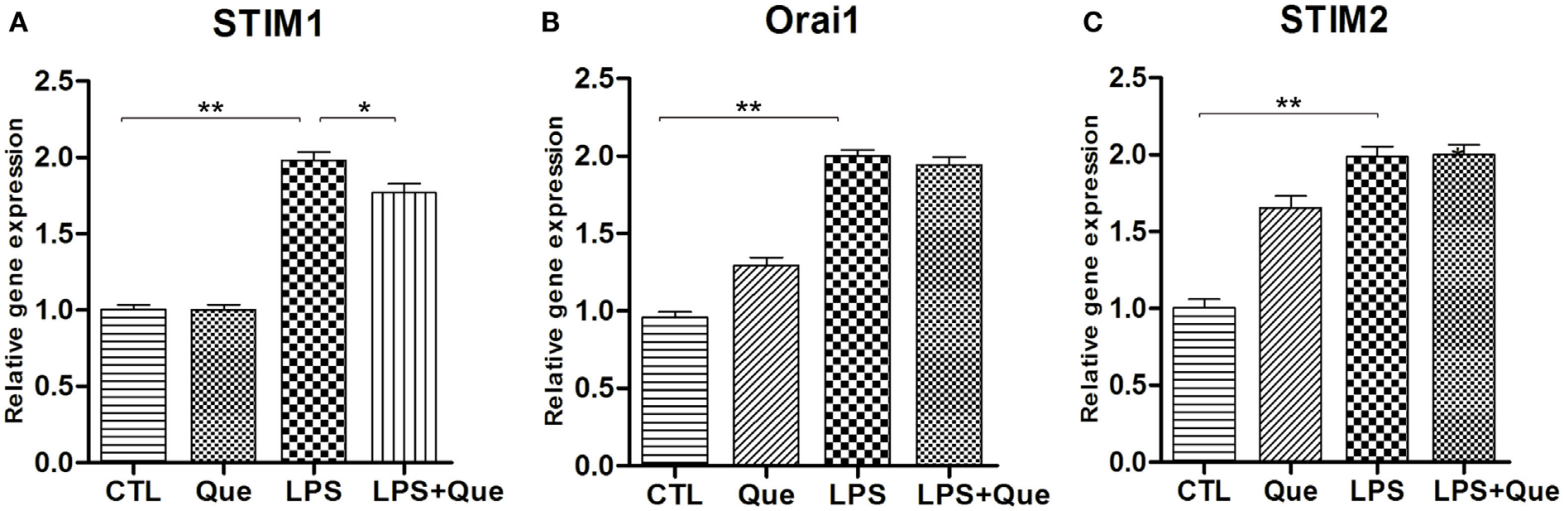
**Effect of Que on expression of SOCE channel proteins in N9 cells exposed to LPS**. Quantification of qRT-PCR showed **(A)** STIM1 mRNA, **(B)** Orai1 mRNA, and **(C)** STIM2 mRNA in N9 cells exposed to LPS with or without 24 h of Que (10 μM) pretreatment. Values are normalized to GAPDH, a loading control. All data are means ± SEM. *p < 0.05, and **p < 0.01 vs. indicated group.

In order to identify the SOCE channels which can be regulated by Que treatment in microglial cells, qRT-PCR was performed to analyze the mRNA levels for SOCE channel proteins STIM and Orai. These SOCE channel proteins were chosen because the interaction of STIM on ER and Orai1 on PM was essential for SOCE activation (Luik et al., 2006). It was found that LPS-exposed cells displayed a significant increase in STIM1 (Figure 4A), STIM2 (Figure 4C), and Orail (Figure 4B) expression as compared to controls (CTLs). Que pretreatment significantly reduced the upregulation of STIM1 (Figure 4A), but produced no effect on Orai1 and/or STIM2 expression (Figures 4B,C).

### Que Inhibits the Translocation of NF-kB p65 in LPS-Activated Microglial Cells

**Figure 5:**
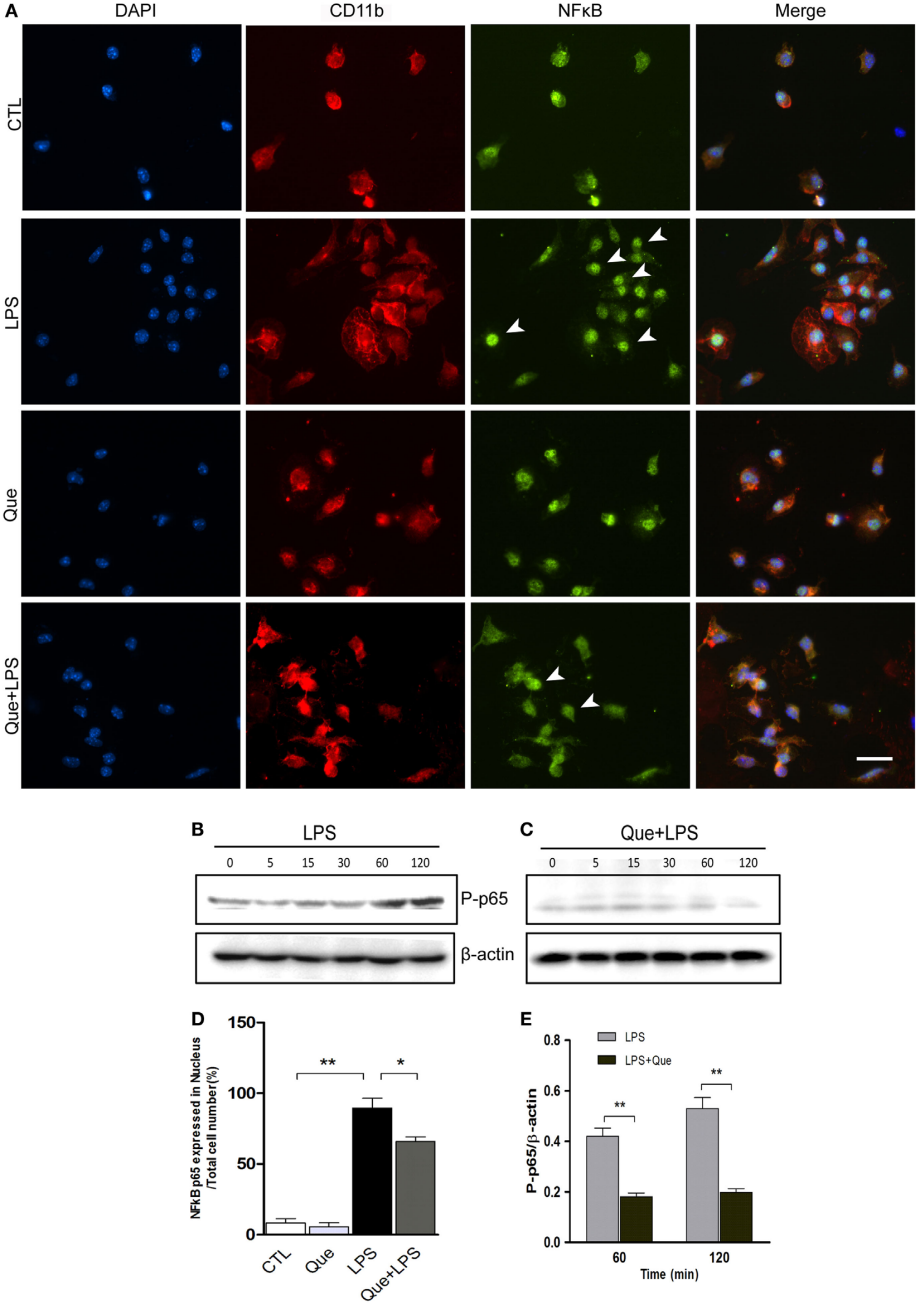
**Effect of Que on activation of NF-κB in N9 cells induced by LPS**. **(A)** Representative double IF staining of NF-κB p65 (green) and CD11b (red) in N9 cells exposed in LPS with or without Que (10 μM) pretreatment, nuclei were stained with DAPI (blue). Note: the arrow indicated positive staining in the nucleus. Scale bars: 50 μm. **(D)** Quantification of the relative ratio of nucleus NF-κB p65 in the indicated groups. Note: LPS significantly increased the ratio of nucleus NF-κB p65, which could be reduced by Que pretreatment. **(B,C)** Western blot analysis of phosphorylated NF-κB p65 in N9 cells exposed in LPS with or without Que pretreatment at indicated time points. β-actin was used as a loading control. Note: the phosphorylated NF-κB p65 level was increased since 60 min after LPS treatment, and this pattern was attenuated by Que. **(D,E)** Quantification of the Western blot showing phosphorylated levels of NF-κB p65 in N9 cells exposed to LPS with or without Que pretreatment. Values are mean ± SEM. *p < 0.05 and **p < 0.01 vs. indicated group.

To further investigate the downstream mechanism of Que inhibition effect on N9 cell activation, we examined alteration of nuclear factor NF-κB activation, which has been implicated in LPS-induced microglial activation (Fan et al., 2015). Immunofluorescence staining revealed that the NF-κB p65 subunits were mainly expressed in cytoplasm and were barely detectable in the nucleus of control (CTL) and Que-treated groups (Que). However, NF-κB p65 subunits were intensively expressed in nucleus after LPS treatment (Figure 5A). However, Que pretreatment significantly reduced the translocation of p65 to nuclei when exposed to LPS (Figures 5A,D). Furthermore, we also examined the effect of Que on phosphorylation of NF-κB p65, a feature of NF-κB activation. The results showed that the phosphorylated level of p65 was significantly increased from 60 min after LPS stimulation; however, Que pretreatment could attenuate this pattern (Figures 5B,C,E). Together, these data indicate that Que can inhibit NF-κB activation in N9 cells induced by LPS.

## Discussion

In the last decade, structural and functional changes in glial cells have been becoming a major focus of interest in the research of SZ (Zheng et al., 2008). In our present study, we demonstrated that microglial activation was associated with the impairment of myelin repair in chronic CPZ-induced demyelination mouse model. In addition, we demonstrated that the atypical APD Que can inhibit the process of microglial activation and promote myelin repair. We further found that Que can suppress the abnormal STIM1-mediated elevation of [Ca^2+^]*_*i*_* and inhibit the activation of NF-κB pathway in LPS-induced microglial cultures. As a result, the inhibitory effects of Que on microglial activation may have important implications for its therapeutic application in SZ.

Cuprizone or biscyclohexnaone oxalyldihydrazone is a ­copper chelator that selectively damages oligodendrocytes. As such, it is a model that has been widely used to induce demyelination. Interestingly, the CPZ-induced demyelination mouse model is also accepted as a SZ model, in which many cognitive functions or behavioral alterations associated with SZ patients are mapped (Makinodan et al., 2009; Xu et al., 2009; Xu et al., 2010; Xu et al., 2011; Praet et al., 2014). Que is currently used in the clinical treatment of SZ. Previous studies indicated that it prevents CPZ-induced white matter pathology and behavioral abnormalities in a short-term feeding (Zhang et al., 2008; Xu et al., 2010; Chandran et al., 2012). To understand the mechanism that may underlie the progression of white matter abnormality to a chronic state, we used the chronic CPZ treatment mouse model, in which the long-term (12 weeks) feeding of CPZ induced a series of demyelination/remyelination episodes, leading to the formation of chronic lesion in white matter (Matsushima and Morell, 2001). We found that long-term CPZ treatment induced serious demyelination and persistent microglial activation even after CPZ was withdrawn. These findings indicate that microglial activation may cause impairment of remyelination, since some cytokines such as INF-γ or Interleukin-1 (IL-I) was found to inhibit oligodendroglia differentiation (Vela et al., 2002; Mana et al., 2006). The proper modulation of activated microglia, however, has been shown to be an integral step in the promotion of CNS remyelination by facilitating the increased production of neurotrophins or decreasing chemokine expression (Butovsky et al., 2006; Emmetsberger and Tsirka, 2012; Zhou et al., 2015). In our study, we demonstrate that Que treatment during the recovery period can attenuate microglial activation and enhance myelin repair. Our research findings are also supported by other researchers that have also shown the beneficial effects of Que in regard to remyelination (Zhang et al., 2012). The findings presented by these researchers suggest that the ability of Que to inhibit microglial activation may represent an important mechanism underlying its effectiveness at promoting remyelination. Normally, cytokines are virtually undetectable in the CNS. As such, when cytokines and chemokines are found in the CNS, it is usually indicative of clinical conditions involving demyelination (Schmitz et al., 2007; Puntambekar et al., 2015), brain trauma (Tasker, 2006), and/or mental disorders such as SZ (Potvin et al., 2008). Therefore, our current research further suggests that manipulating the microglial activation may be a key step in facilitating myelin repair that can be pursued as a novel treatment approach for SZ and/or other white matter disorders.

In regard to the specific mechanisms by which Que affects microglia, we demonstrate that Que can modulate STIM1-mediated intercellular calcium elevation induced by LPS. As an important second message molecule, elevation of cytosolic Ca^2+^ has an essential role in microglial activation, including proliferation, migration, ramification, and release of proinflammatory cytokines and neurotrophins such as brain-derived neurotrophic factor (BDNF) (Mizoguchi et al., 2014). Although Ca^2+^ entry across the plasma membrane (PM) is mediated by various channels, SOCE channels have been identified as the prevalent Ca^2+^ entry mechanism in non-excitable cells, such as microglia (Laskaris et al., 2015). SOCE is controlled by stromal interaction molecule (STIM1 or STIM2) on the membrane of ER and Orai1 on PM. STIM1 acts as ER Ca^2+^ sensor (Liou et al., 2005; Roos et al., 2005) and as such can activate Ca^2+^ release-activated Ca^2+^ (CARC) channels *via* interaction with Orai (Moccia et al., 2015). It has been shown that STIM1 is required for SOCE in immune cells and loss of function or null mutations in human STIM1 gene stops Ca^2+^ influx in T cells resulting in immunodeficiencies in affected patients (Feske, 2009; Fuchs et al., 2012). Previous study also showed that Orai1–STIM1 interaction on PM can mediate Ca^2+^ influx, thereby regulating cytokine release in microglia (Sun et al., 2014; Laskaris et al., 2015). Pharmacological inhibitors and knockdown experiments using siRNA for Orai1 and STIM1 revealed that the inhibition of Ca^2+^ influx through SOCE diminished the secretion of cytokines, TNF-α and IL-6 (Heo et al., 2015). In the present study, we demonstrate that Que did not modulate basal level of cytosolic Ca^2+^; however, it can ameliorate LPS- and Tg-induced elevation of Ca^2+^ influx *via* neutralizing the upregulation of STIM1. Based on our understanding in this area, it is the first time that the regulatory effect of Que on the SOCE channel that is involved in microglial activation is shown. However, the exact molecular mechanism how Que modulates SOCE still requires further investigation. Our research findings suggest that manipulating SOCE may represent a novel approach to attenuate the chronic disease progression associated with SZ. In addition, our research also suggests that STIM1 is a novel regulatory target for neutralizing microglial activation that may be advantageous in the prevention of progression of various neurodegenerative diseases or mental illness.

Following elevation of cytosolic Ca^2+^, several downstream signaling pathways, such as NF-κB activation, are primarily responsible for microglial activation (Chauhan et al., 2014). NF-κB is a transcription factor that can be involved in multiple cell behaviors such as cell differentiation, survival, apoptosis, as well as immune and inflammatory response (Li and Verma, 2002; Vallabhapurapu and Karin, 2009). It has been reported that in AD or ischemia models, Que can inhibit activation of NF-κB in neurons (Bi et al., 2009; Zhao et al., 2014). Moreover, in an AD mouse model, Que was shown to attenuate glial activation and reduce the release of proinflammatory cytokines *via* inhibition NF-κB pathway (Zhu et al., 2015). In the present study, we also demonstrate that Que inhibited the translocation of NF-κB p65 and decreased the phosphorylated levels of NF-κB p65 subunits induced by LPS, indicating that the inhibition effect of Que on microglial activation may due to its effect to suppress NF-κB activation. Overall, it is likely that Que can maintain the intercellular calcium homeostasis by modulating STIM1 expression and subsequently inhibit NF-κB-dependent microglial activation.

In summary, within CPZ-induced chronic demyelination mouse model in conjunction with an *in vitro* system involving LPS-induced microglial activation cultures, we demonstrate that the atypical APD Que can inhibit microglial activation by neutralizing abnormal STIM1-mediated intercellular calcium homeostasis. In addition, our results also suggest that the ability of Que to inhibit microglial activation may also promote myelin repair. Our research findings identify a novel approach of manipulating specific calcium channels to regulate microglial activation that contributes to the underlying pathogenesis of SZ.

## Author Contributions

HW, HL, and LX designed the study. HW, SL, YT, XW, YH, and HL acquired and analyzed the data. CL, JK analyzed the data. HW, HL, MN, and LX wrote the article, which all other authors reviewed. All authors approved the final version for publication.

## Conflict of Interest Statement

Lan Xiao and Hanzhi Wang declare having received grant funding for this work as above. The remaining authors have nothing to declare.
